# The Emerging Nexus of Active DNA Demethylation and Mitochondrial Oxidative Metabolism in Post-Mitotic Neurons

**DOI:** 10.3390/ijms151222604

**Published:** 2014-12-05

**Authors:** Huan Meng, Guiquan Chen, Hui-Ming Gao, Xiaoyu Song, Yun Shi, Liu Cao

**Affiliations:** 1 Key Laboratory of Medical Cell Biology, Ministry of Education, China Medical University, Shenyang 110001, China; E-Mail: xysong@mail.cmu.edu.cn (X.S.); 2 Key Laboratory of Model Animal for Disease Study, Ministry of Education, Model Animal Research Center, Nanjing Biomedical Research Institute, Nanjing University, Nanjing 210061, China; E-Mails: chengq@nicemice.cn (G.C.); gaohm@nicemice.cn (H.-M.G.); shiyun@nicemice.cn (Y.S.)

**Keywords:** active DNA demethylation, mitochondrial oxidative metabolism, TET (ten-eleven translocation) methylcytosine dioxygenases, post-mitotic neurons, neurodegeneration

## Abstract

The variable patterns of DNA methylation in mammals have been linked to a number of physiological processes, including normal embryonic development and disease pathogenesis. Active removal of DNA methylation, which potentially regulates neuronal gene expression both globally and gene specifically, has been recently implicated in neuronal plasticity, learning and memory processes. Model pathways of active DNA demethylation involve ten-eleven translocation (TET) methylcytosine dioxygenases that are dependent on oxidative metabolites. In addition, reactive oxygen species (ROS) and oxidizing agents generate oxidative modifications of DNA bases that can be removed by base excision repair proteins. These potentially link the two processes of active DNA demethylation and mitochondrial oxidative metabolism in post-mitotic neurons. We review the current biochemical understanding of the DNA demethylation process and discuss its potential interaction with oxidative metabolism. We then summarise the emerging roles of both processes and their interaction in neural plasticity and memory formation and the pathophysiology of neurodegeneration. Finally, possible therapeutic approaches for neurodegenerative diseases are proposed, including reprogramming therapy by global DNA demethylation and mitohormesis therapy for locus-specific DNA demethylation in post-mitotic neurons.

## 1. Introduction

The variable patterns of DNA modification, in particular DNA methylation patterns in mammals, have been linked with a number of physiological processes, including normal embryonic development and disease pathogenesis [1,2,3,4]. Different types of DNA methylation have been discovered in animals, including 5-methylcytosine (5mC) and the recently discovered 5-hydroxymethylcytosine (5hmC), 5-formylcytosine (5fC) and 5-carboxylcytosine (5caC) [5,6,7]. Patterns of CpG methylation (5mC) catalysed by DNA cytosine methyltransferases (DNMTs) can be relatively stable in terminally differentiated cells. However, identification of 5hmC and its derivatives, 5fC and 5caC in post-mitotic neurons suggests that ten-eleven translocation (TET) family enzymes can mediate dynamic reprogramming of DNA methylation (both globally and site specifically) [8,9]. Thymine DNA glycosylase (TDG)-mediated base excision of 5fC and 5caC may facilitate their replacement by unmodified cytosine in a potential cycle of methylation and demethylation [8,10]. The presence of methylated CpG dinucleotides can alter the accessibility of DNA-binding proteins to chromatin, in particular providing high-affinity targets for the binding of methyl-CpG binding proteins (MeCPs) [11]. Changes in the DNA methylation profile of somatic cells can indirectly alter histone modifications by changing the accessibility of histone-modifying complexes that are preferential targets of either modified or unmodified DNA [12,13,14]. In this view, active removal of DNA methylation in a genome-wide or gene-specific manner may impact on a variety of cellular processes through its differential affinity for nuclear complexes. In support of this, dynamic modifications of 5mC and 5hmC have been identified in mouse embryonic stem cells (mESCs), neuronal progenitor cells (NPCs), and adult mouse brain tissue [11]. Increasing evidence suggests that the TET dioxygenase enzymes and DNA modifications involved in the active removal of DNA methylation may be influenced by oxidative metabolites and oxidizing agents in non-dividing cell types such as post-mitotic neurons, linking mitochondrial oxidative metabolism to active DNA demethylation. In this review, we begin by introducing model mechanisms of active removal of DNA methylation. We then summarise the possible associations between active DNA demethylation and mitochondrial oxidative metabolism from multiple levels, including TET methylcytosine dioxygenases-mediated oxidation and oxidative DNA products such as 5-hydroxymethyluracil (5hmU). Some recent advances are reviewed in detail, in particular the emerging role of TET dioxygenases in memory formation and reversal learning. Finally, we briefly discuss the potential role of DNA demethylation in neurodegeneration, and then propose possible therapeutic approaches with respect to DNA demethylation for designing novel treatments for degenerative post-mitotic neurons.

## 2. DNA Methylation Is Stable and Reversible

Chromatin-based processes of transcription, DNA replication and DNA repair depend on epigenetic signalling that flexibly employs reversible modifications of chemically stable marks in DNA and histone proteins. The stability of these epigenetic marks varies widely, depending on the forms of covalent modifications and the chemical enzymes involved. Deacetylation, dephosphorylation and deubiquitylation at Lys (lysine), Ser (serine) and Arg (arginine) residues of histone proteins use less energy and are easier to modify, because they form ester or amide bonds that can be removed by hydrolytic enzymes [15]. In contrast, demethylation at cytosine DNA residues and Lys of histone proteins requires modification of the inert methyl groups at C–N and C–C bonds, which are difficult to remove and are therefore thought as long-lived epigenetic marks [15]. Experimental evidence of lysine demethylase reveals one-step active reversal of histone modifications by corresponding enzymes [16]. Unlike histone modifications, multi-step processes are potentially involved in active removal of DNA methylation in mammals.

Mammalian DNA methylation occurs at the 5-position of cytosine and this epigenetic modification is found predominately in CpG dinucleotides [4]. In general, DNA methylation is established by the *de novo* DNA methyltransferases DNMT3A and DNMT3B and its patterns are maintained by the maintenance DNA methyltransferase DNMT1. The patterns of DNA methylation are relatively stable and maintained through cell generations. In addition to this classical maintenance model, from a developmental view, mounting experimental evidence has underscored the influence of DNA demethylation on DNA methylation patterns both by passive mechanisms occurring at replication and by active processes after replication [17,18,19]. Active DNA demethylation can act as a powerful mechanism to dynamically regulate gene expression. The active demethylation targeting specific loci is often coupled with base excision repair (BER) proteins, TET dioxygenases, deaminases and/or DNMTs in a cyclic manner or in response to certain stimuli [20,21,22,23,24].

**Figure 1:**
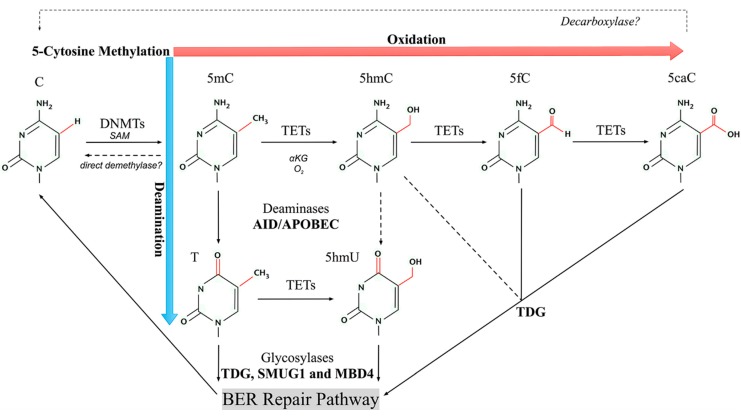
Model pathways of active DNA demethylation. DNA cytosine methyltransferases (DNMTs) with co-factor *S*-adenosyl methionine (SAM) establish and maintain 5-methylcytosine (5mC) from cytosine (C). A direct demethylase was hypothesised but has not been identified to date. The ten-eleven translocation (TET) family of dioxygenases can oxidize 5mC to generate 5-hydroxymethylcytosine (5hmC), 5-formylcytosine (5fC) and 5-carboxylcytosine (5caC). 5fC and 5caC can be removed by glycosylases such as thymine DNA glycosylase (TDG). Alternatively, a decarboxylase that may convert 5caC to C is speculated. 5mC and 5hmC can be deaminated by AID/APOBEC deaminases to form thymine (T) and 5-hydroxymethyluracil (5hmU), respectively. In addition, T can be oxidized by TETs to produce 5hmU and both T and 5hmU can be excised by glycosylases such as TDG, single-strand-selective monofunctional uracil-DNA glycosylase 1 (SMUG1) and methyl-CpG binding protein 4 (MBD4).

Mechanisms of active removal of DNA methylation have been proposed (Figure 1), which are supported by *in vitro* and *in vivo* experimental evidence [17]. The first model involved direct removal of the methyl group, inspired by the identification of the four bifunctional DNA glycosylases responsible for processing 5mC in CpG and non-CpG contexts in plants [20]. In mammals, reproduction of the suspected glycosylases methyl-CpG binding protein (MBD4) and thymine DNA glycosylase (TDG) as direct demethylases has not been widely accepted by the scientific community to date. However, it is noteworthy to mention that the monofunctional glycosylases MBD4 and TDG indeed show weak enzyme activity for 5mC *in vitro* [25]; therefore, it is still possible that their co-factors or post-translational modifications *in vivo* may enhance the catalytic efficiency. However, recent findings have favoured multi-step demethylation through deamination or oxidation or a combination of both, followed by glycosylases and the BER repair pathway.

The idea of more favourable substrates transformed from 5mC for subsequent excision led to the second model of deamination route and the third model of oxidation route. In the deamination model, the cytidine deaminases AID/APOBEC or the methyltransferases Dnmt3a/b have been proposed to deaminate 5mC to T, generating a T:G mismatch that can be recognised and excised by the glycosylases MBD4 or TDG [21,22,26]. Following the BER repair pathway, T can be replaced by cytosine eventually. This deamination model route was tested successfully at the specific gene locus on the *pS2*/*TFF1* promoter that is responsive to oestrogen stimuli [22]. It was also validated in a zebrafish embryo model using an exogenous methylated DNA reporter [26]. In the former report, DNA demethylation at the *pS2*/*TFF1* promoter involves 5mC deamination through the methyltransferases Dnmt3a/b and a RNA helicase p68 [22]. In contrast, AID deaminase and an auxiliary factor Gadd45 were implicated in the case of deamination-coupled demethylation in zebrafish embryos [26]. However, the specificity of the AID/APOBEC deaminase family is single-strand-selective, and this was perceived as the main challenge of this possible mechanism [17,27]. The third oxidation model of active DNA demethylation was supported by a number of key findings discovered recently. This model is based on the discovery of TET dioxygenases and the 5mC oxidation product 5hmC [6,28]. TET proteins can iteratively oxidize 5hmC to 5fC and 5caC, followed by TDG excision, which completes the demethylation cycle [29,30]. Alternatively, a putative 5caC decarboxylase is hypothesised, which remains to be identified [17]. This oxidation route is currently most plausible because it has been supported by both *in vitro* and *in vivo* evidence and is widely accepted. However, the energy-consuming and lengthy processes do not appear to fit the rapid and cyclic case of active removal of DNA methylation.

The fourth model came from a combination of the processes of 5mC oxidation followed by deamination, or *vice versa*, both of which generate a common 5hmU intermediate. 5hmU is pro-mutagenic, and 5hmU mismatched with guanine (G) can be efficiently processed by either TDG or MBD4 or single-strand-specific monofunctional uracil DNA glycosylase (SMUG1) [25]. However, the 5mC–5hmC–5hmU model was challenged by the fact that the deamination of 5hmC by AID/APOBEC deaminases was not detected in a subsequent systematic biochemical study [31]. In contrast, spontaneous deamination of 5mC to T was examined and is currently widely accepted in various biological models [25]. More recently, using isotopologs, Pfaffeneder *et al.* [32] found that the origin of the majority (70%–80%) of hmU in mESCs was not from endogenous reactive oxygen species (ROS) or deamination of hmC; instead, the Tet1 catalytic domain was responsible for oxidation of T to hmU that is paired with A (adenine) in mESCs. Thus, the alternative 5mC–T–5hmU route appears more appropriate for this combinatory model, at least in mESCs. Moreover, in the same study, low but substantial levels of deamination of hmC to hmU were detected in mouse *Tdg*^−/−^ stem cells reconstituted by a catalytically inactive Tdg, which helps to form a complex with the deaminase AID. This represented a paradoxical case in contrast to the AID/APOBEC specificity study [17,27], because the reforming AID complex appears capable of converting hmC to hmU in double-stranded DNA [32].

Collectively, mammalian systems may have multiple pathways to perform active DNA demethylation to deal with different scenarios. These pathways provide multiple levels of potential targets on which metabolic oxidation intermediates and ROS stress may exert their influence, including methylcytosine dioxygenases TETs and their O_2_ dependent dioxygenation and the DNA bases vulnerable to oxidative damage such as the oxidative derivatives of methylated cytosine in the context of CpG dinucleotide.

## 3. TET (Ten-Eleven Translocation) Dioxygenases Link Metabolic Intermediates to Active DNA Demethylation

**Figure 2:**
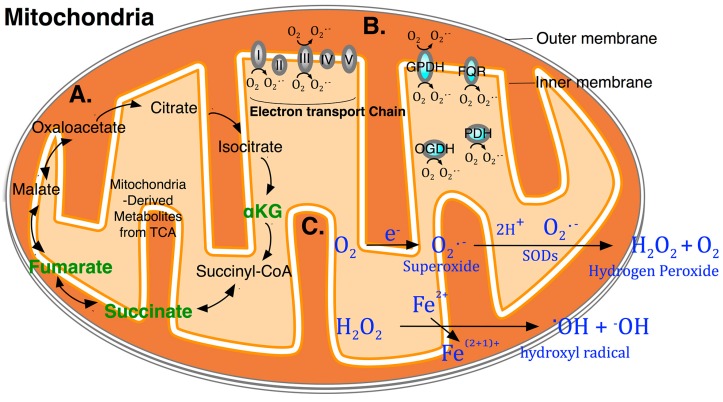
Mitochondria produce oxidative metabolites and reactive oxygen species (ROS). (**A**) α-ketoglutarate (αKG), its oxidized and decarboxylated product succinate, and its further oxidation product fumarate (all labelled bold green) are the key metabolites generated from the mitochondrial tricarboxylic acid (TCA) cycle; (**B**) Multiple sites generate superoxide anion (O_2_^•−^) in mitochondria [36]. Complex I and III of the electron transport chain as well as mitochondrial GPDH, FQR, OGDH and PDH are capable of generating O_2_^•−^ on the matrix side of the mitochondrion. In addition, complex III and GPDH can generate O_2_^•−^ in the inner mitochondrial membrane space. GPDH, glycerol 3-phosphate dehydrogenase (also known as GPDM); OGDH, 2-oxoglutarate dehydrogenase; PDH, pyruvate dehydrogenase; FQR, electron transfer flavoprotein-ubiquinone oxidoreductase; (**C**) Representative endogenous sources and processes of ROS. Upper, enzyme-mediated conversion of O_2_^•−^. O_2_^•−^ can be either a by-product of respiration or oxidation product of NADPH oxidase-mediated reduction. Subsequently, superoxide dismutase (SOD) can convert O_2_^•−^ into hydrogen peroxide (H_2_O_2_). Lower, Fe^2+^/Fe^(2+1)+^ as a reduced and oxidized transition metal ion, mediates the generation of the hydroxyl radical (^•^OH) from H_2_O_2_.

TET family proteins are AlkB-like Fe(II)/α-ketoglutarate-dependent dioxygenases. Recent studies have demonstrated that the TET proteins TET1, TET2, and TET3 can oxidize 5mC to generate 5hmC, 5fC and 5caC, mediating DNA demethylation by iterative oxidation in cooperation with the BER repair pathway [17]. For TET dioxygenases, there are two stages constituting the Fe(II)/α-ketoglutarate (αKG)-dependent oxidation of 5mC. The first stage is the dioxygen activation stage, where two electrons from Fe(II) and two from αKG contribute to form a peroxo bridge and then produce Fe(IV)-oxo intermediate [33]. In the second stage of substrate oxidation, the C–H bond of 5mC is oxidized by the reactive species Fe(IV)-oxo, and Fe(II) is restored from Fe(IV) as the catalyst [17]. In this chemical reaction cycle, succinate, the oxidized and decarboxylated form of αKG, receives one oxygen atom of the dioxygen molecule and the oxidized product 5hmC incorporates another oxygen atom. It is noteworthy that αKG, succinate and its further oxidation product fumarate are the key metabolites of the mitochondrial tricarboxylic acid (TCA, or Krebs) cycle (Figure 2A). Succinate, fumarate and the other oncometabolite 2-hydroxyglutarate (2-HG) share structural similarities with αKG, thereby functioning as competitors of αKG to inhibit the demethylation activity of TET dioxygenases. The association between metabolic intermediates from the TCA cycle as co-factors and the enzyme activity of oxygen-dependent dioxygenases has linked the oxidative metabolism to active DNA demethylation. In mice endogenously expressing the IDH1(R132H) mutant in the central nervous system (CNS), the mutant *Idh1* gene resulted in approximately 400–500-fold elevation in 2-HG levels, while αKG decreased modestly [34]. Excessive 2-HG levels inhibited dioxygenase activity, which was associated with abnormal phenotypes in the mutant mouse CNS, including defective angiogenesis and an altered microenvironment [34]. Increased stability of hypoxia-inducible transcription factor HIF1α and its endoplasmic reticulum (ER) accumulation were found in the brain of these mutant mice, which appeared to be a cause of the failure of collagen deposition along blood vessels in the CNS of IDH1(R132H) mutants [34]. Similar to TETs, HIF1α also belongs to the dioxygenase family and plays a key role in the cellular response to hypoxia [35]. The stability of HIF1α is regulated by the prolyl hydroxylase domain-containing protein (PHDs), which in principle requires αKG as a cofactor and is also a potential target influenced by 2-HG competition. Collectively, involvement of αKG and its metabolic competitors in dioxygenase-dependent oxidation establishes the nexus between mitochondrial oxidative metabolism and active DNA demethylation, which may be associated with cellular pathways such as HIF1α-dependent autophagy. Metabolic ROS and oxidizing agents may also target active DNA demethylation at multiple levels; in principle, involvement of ROS-induced oxidative stress could employ both mechanisms of genotoxic DNA damage that results in oxidative derivatives of methylated cytosine and non-genotoxic signalling that is capable of regulating the activity or specificity of the protein enzymes involved.

## 4. ROS (Reactive Oxygen Species) and Oxidizing Agents Modify Methylated Cytosine Derivatives

Oxidative stress describes a systemic imbalance of excessive production of ROS over reduction of reactive intermediates or resulting damage in antioxidant defence or repair. ROS are small molecules originating from molecular oxygen (O_2_), mostly in the forms of superoxide anion (O_2_^•−^) and hydrogen peroxide (H_2_O_2_). Mitochondria are a main source of cellular ROS, where O_2_ is reduced by one electron to O_2_^•−^ in the major sites of complexes I and III of the electron transport chain [36] (Figure 2B). In addition to depositing O_2_^•−^ into the mitochondrial inner membrane matrix, site IIIQo on complex III and glycerol 3-phosphate dehydrogenase (GPDH, also known as GPDM) can produce O_2_^•−^ into the intermembrane space [37] (Figure 2B). Subsequently, O_2_^•−^ is converted to H_2_O_2_ by superoxide dismutases (SODs) within both mitochondria and cytosol [38] (Figure 2C, upper). In addition, there are other endogenous (e.g., cytosolic enzymes NADPH oxidases and phagocytes) [36] and exogenous sources of ROS (e.g., metal ions, chemotherapeutic and chemopreventive agents, and ionizing radiation) [39]. Virtually all cellular macromolecules including proteins, lipids, RNA, and DNA are subject to ROS attack, which attracted extensive investigations of the roles of ROS in terms of their harmful effects in past decades.

Metabolically produced H_2_O_2_ can react with the reduced redox-active metal ions ferrous and cuprous by a reaction termed Fenton reaction [40], which generates a hydroxyl radical (^•^OH) (Figure 2C, lower). ^•^OH is a highly reactive radical and its reaction essentially occurs at the site of generation. The structure of DNA macromolecules is particularly attractive for labile metal ions; thus, the locally generated ^•^OH by Fenton-like reactions is believed to attack vulnerable DNA bases in a site-specific manner. In addition, ionizing radiation provides an indirect source of ^•^OH by the radiolysis of water molecules. In terms of DNA macromolecules, cytosine and its derivative 5mC and thymine (T, also known as a deamination product of 5mC) are prominent targets of ^•^OH radical reactions [41].

^•^OH preferentially adds to T, cytosine, and 5mC; in particular, it can abstract an H-atom from the methyl group [41]. This H-atom abstraction results in the intermediates 5-(uracilyl)methyl radical and H_2_O_2_ in the case of T and the corresponding peroxyl radical after O_2_ addition. Subsequent reduction and competitive dehydration generate 5hmU and 5-formyluracil (5fU). Similarly, the ^•^OH-mediated decomposition of cytosine generates intermediate compounds of uracil hydroperoxides and a cyclic endoperoxide, which subsequently formats more than 30 labile and stable products, including 5-hydroxycytosine (5hC) and 5-hydroxyuracil (5hU). The precise contribution of oxidative products generated from ionizing radiation or Fenton reaction in isolated and cellular DNA remains largely elusive [41]. Of particular interest, the cytosine derivatives 5mC and T, as well as their ROS-mediated oxidation products such as 5hmU, have been extensively identified in the aforementioned model pathways of active DNA demethylation [5,6,18,42], implying that ROS and oxidizing agents may have an important role in this critical process, at least in part, by modifying methylated cytosine derivatives.

## 5. Active DNA Demethylation in Post-Mitotic Neurons

Post-mitotic neurons are a paradigm model to study active DNA demethylation mechanisms. Most of these neurons are long-lived, and free of DNA replication. Therefore, any DNA demethylation event in post-mitotic neurons is likely to follow active rather than passive pathways. In the fully matured neurons that are in the post-mitotic state, a number of physiological or pharmacological stimuli such as membrane depolarization, hormonal treatment, and drug perturbation are able to induce substantial changes in DNA methylation profiles [43]. DNA demethylation induced by membrane depolarization has been revealed in elegant experiments studying the promoters of brain-derived neurotrophic factor (*Bdnf*) and fibroblast growth factor-1 (*Fgf-1*) [44,45,46]. Two earlier studies reported that membrane depolarization induced by KCl in primary cortical neuron cultures was sufficient to initiate active demethylation at the CpG sites of proximal promoter regions of exon III or IV of the *Bdnf* gene, which corrected well with the activity-dependent transcription of the corresponding *Bdnf* exons [44,45]. Consistently, specific DNA demethylation at above regulatory regions of *Bdnf* and *Fgf-1* was shown to respond to electro-convulsive treatment (ECT) induction [46].

Physiological stimuli of hormonal treatments or maternal nutrition can also induce changes in the CpG methylation profile at the glucocorticoid receptor (*GR*) promoter in rat CNS. In rat offspring that received high levels of maternal care, decreased CpG methylation was observed in the *GR* promoter regions in the hippocampus, which was subsequently associated with alterations in the chromatin state and binding of *GR* transcriptional factors [47]. In post-mitotic hepatic cells, glucocorticoid-induced CpG demethylation has been found at the glucocorticoid responsive sequence, and such alteration was considered to be an epigenetic cellular memory to prime the promoter for the subsequent stimuli [43,48]. These findings suggest that direct hormonal stimuli or hormone-associated behavioural or environmental changes, such as maternal care, may influence particular hormonal responses by active DNA demethylation in a positive feedback manner for *GR* expression. The maternal effects of epigenetic marking, *GR* expression and stress responses can be reversed by the histone deacetylase (HDAC) inhibitor trichostatin A (TSA) [47]. In principle, inhibition of HDAC may trigger the activation of chromatin and thus expose the accessible chromatic regions to transcriptional factors, subsequently inducing DNA demethylation [49]. It remains unknown what precise processes and components of DNA demethylation are involved in such experimental paradigms and if any DNA methyltransferase is participating in the activities. In a different model of hypoxic brain injury using mice subjected to mild ischemic stress, increased *in vivo* levels of DNA methylation were observed in mice with augmented brain injury, which was associated with a substantial increase in the catalytic activity of Dnmt1 [50]. Subsequent inhibition of DNA methylation by 5-aza-2′-deoxycytidine (5-AzadCyD, 5-Aza-CdR, decitabine) and TSA enhanced the survival rate of these mice subjected to mild ischemic stress [50]. It appears that active DNA demethylation plays an important role in modifying dynamic patterns of DNA methylation profiles in post-mitotic neurons, which potentially regulates critical brain functions in response to a number of physiological or pharmacological stimuli.

## 6. TET Methylcytosine Dioxygenases in Memory Formation and Reversal Learning

Dynamic DNA methylation has been implicated in learning and memory, particularly in terms of alterations in neuronal gene expression contributing to experience-dependent plasticity and behaviour [51,52,53,54,55]. Active DNA demethylation, which potentially modifies DNA methylation patterns in post-mitotic neurons, has attracted considerable attention in brain function. Recent studies have suggested that a number of key enzymes involved in the active removal of DNA methylation, including TET dioxygenases, are associated with memory formation and reversal learning such as fear extinction [56,57,58,59,60] (Table 1).

**Table 1:** Function of mouse TET methylcytosine dioxygenases.

Tet Dioxygenases	Transcription in Adult Mouse Brain [61]	Known DNA Substrates	Major Mouse Phenotypes Partially from Mouse Genome Informatics (MGI)
**Tet1**	Cerebellum: medium Cortex: low Hippocampus: low	5mC to 5hmC T to 5hmU [32] 5hmC to 5fC 5fC to 5caC	Knockout mice are viable, fertile and grossly normal. Some mutant mice have mild embryonic growth retardation, decreased body size and small litters [ 62]. One line of knockout mice exhibits abnormal hippocampal long-term depression and impaired memory extinction [58]. Another line of knockout mice exhibits impaired hippocampal neurogenesis accompanied by poor learning and memory [59].
**Tet2**	Cerebellum: medium Cortex: medium Hippocampus: medium	5mC to 5hmC 5hmC to 5fC 5fC to 5caC	Knockout mice evolved to a wide spectrum of lethal myeloid malignancies [63]. Conditional knockout of hematopoietic compartment exhibits increased stem cell self-renewal and myeloid transformation [64–66].
**Tet3**	Cerebellum: high Cortex: high Hippocampus: high	5mC to 5hmC 5hmC to 5fC 5fC to 5caC	Conditional knockout mice show impaired reprogramming of the paternal genome, resulting in reduced embryo viability. Female germ-line knockout mice show severely reduced fecundity and some of their heterozygous mutant offspring have increased developmental failure [67]. Cortical knockdown in mice shows impairment in fear extinction memory [57].

The 5mC oxidation product 5hmC and all three TET proteins are highly enriched in the adult brain [5,68], indicating that they may have important roles in brain function. Guo *et al.* [60] initially reported a Tet1-mediated hydroxymethylation in response to neuronal activity in the adult brain. In the dentate gyrus (DG) of the adult mouse hippocampus, increased global 5hmC was observed by Tet1 overexpression, which depended on Tet1 catalytic activity [60]. Moreover, the previously identified electroconvulsive shock-sensitive demethylation at a number of promoter regions of *Bdnf* exon IX and *Fgf1b* and its regulated gene expression [46] were completely abolished by Tet1 knockdown [60]. This was the first study to demonstrate the involvement of 5mC dioxygenase Tet1 in neuronal activity-induced DNA demethylation in post-mitotic neurons [60]. In a subsequent study, mice lacking Tet1 were shown to exhibit poor learning and memory, and these phenotypes were linked to down-regulation of several adult neurogenesis-related genes whose promoters were hypermethylated in hippocampal progenitor neurons in the adult brain [59]. Kaas *et al.* [56] further used a spatiotemporal restriction approach to make a TET1 overexpression model in the mouse hippocampus to examine the potential involvement of TET1 in memory formation. They were inspired by the observation that the transcriptional levels of hippocampal Tet1 and global methylation profiles were altered by neuronal activity [56]. Several neuronal genes sensitive to neuronal activity were found to be dysregulated by TET1 overexpression in the dorsal hippocampus, which was associated with the brain function of memory formation [56]. Both catalytically active TET1 and its inactive mutants were capable of mediating regulation of the same set of neuronal memory-associated genes and disrupting the long-term memory formation process after fear conditioning in a similar way [56], suggesting that the catalytic role of TET1 may be dispensable and that attracting other factors may be one likely mechanism in this scenario. The *in vivo* overexpression of TET1 was concomitant with the expression of a number of DNA repair enzymes implicated in active DNA demethylation, including Tdg, Apobec1, Smug1 and Mbd4 [56]. Further investigations are needed to explore the possible involvement of these enzymes in 5mC conversion in post-mitotic neurons.

In another parallel study using Tet1-deficient mice as a model, Rudenko *et al.* [58] reported normal brain size and morphology but a reduction in 5hmC levels in the cortex and hippocampus. These mice were identified to have abnormal hippocampal long-term depression and impaired memory extinction [58]. Tet1-mediated hydroxymethylation was further examined at the promoter loci of several markedly down-regulated neuronal plasticity genes in the cortex and hippocampus of Tet1KO mice [58]. They found an association between promoter CpG hypermethylation and the relatively down-regulated expression of a master regulator neuronal PAS domain protein 4 (*Npas4*) in both naive Tet1KO mice and mice subjected to memory extinction training [58]. Such hypermethylation, presumably caused by the lack of Tet1 catalytic activity, was proposed to be a likely mechanism for suppression of *Npas4* in the subsequent examinations with and without memory extinction training [58]. However, further investigations into hydroxymethylation at gene body and distal regulatory elements are required in this model, because these loci, and not the promoter region, are widely accepted as common Tet1 targeting sites [58]. It is also noteworthy that Tet1KO mice showed impairment of memory extinction but exhibited normal memory acquisition under stronger stimuli such as Pavlovian fear conditioning [58]. One possibility is that other Tet proteins may be revoked by the aforementioned stronger stimulus to compensate the Tet1 inactivation.

More recently, Tet3-mediated 5mC hydroxymethylation was also reported to be required for rapid behavioural adaptation using an experimental paradigm of fear extinction as a model of reversal learning in mice [57]. Unlike Tet1, which showed activity in the hippocampus, Tet3-dependent activity in response to fear extinction training was observed only in cortical neurons [61]. This is consistent with the observation of high expression of Tet3 in the adult cortex. In contrast to Tet1-mediated accumulation of 5hmC within distal regulatory elements and proximal promoters after fear learning, Tet3 was associated with intragenic 5hmC accumulation of non-promoter redistribution in response to extinction training [57]. These data suggest that Tet1 and Tet3 may act differently in terms of gene regulation, stimulus responses and epigenetic landscapes, although all Tets share 5mC oxidation function [57]. Similar to Tet1, the catalytically inactive mutant of Tet3 was found to be capable of inducing some degree of DNA demethylation [57]. The precise mechanisms by which TET dioxygenases exert their roles both catalytically and non-catalytically remain to be determined.

**Figure 3:**
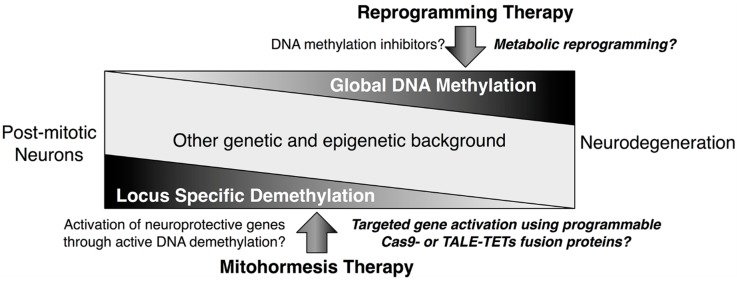
Working model of DNA demethylation therapies for neurodegeneration. Mounting evidence has shown that patterns of DNA methylation are altered in adult post-mitotic neurons in neurodegenerative diseases, which in principle may involve increased global DNA methylation or decreased locus-specific demethylation. The possible reprogramming therapy (**upper**) is proposed using DNA methylation inhibitors or metabolic reprogramming to “reset” the patterns of DNA methylation in progressively degenerative adult neurons. Autonomous events are expected to be driven by a reactivated set of “stemness” genes to form fresh epigenetic marks and a functional neuronal gene network for recovery of some critical brain functions. In contrast, mitohormesis therapy (**lower**) is based on our speculation that the beneficial effects of ROS stress acting on cytoplasmic signalling may at least in part, reactivate a number of neuroprotective genes by triggering nuclear responses through possible pathways of active DNA demethylation or other yet unknown mechanisms. A targeted gene activation approach using programmable Cas9- or transcription activator-like effector (TALE)-TETs fusion proteins may be useful for the locus specific demethylation.

As mentioned above, the mitochondrial metabolites such as αKG, succinate and fumarate and ROS such as ^•^OH may influence active DNA demethylation by altering the catalytic activity of TETs or modifying the oxidative derivatives of methylated cytosine. The oxidation and repair enzymes involved and their DNA substrates have been increasingly implicated in post-mitotic neuronal activities [19]. Considering this, we propose a possible link between active DNA demethylation and mitochondrial oxidative metabolism in a paradigm model of post-mitotic neurons. Given the recurrence of memory and learning disabilities in cases of neurodegeneration, it is not too far afield to speculate that insights into such a nexus may provide novel therapeutic strategies for neurodegenerative diseases (Figure 3).

## 7. Implications for Potential Therapies of Neurodegeneration

Adult post-mitotic neurons may have distinct patterns and characters of epigenetic marks, particularly regarding those of long-lasting DNA methylation in adult neurons that are non-dividing, long-lived and cell fate-perpetuated. Accordingly, the term neuroepigenetics was adopted recently to describe the roles of epigenetic mechanisms in adult post-mitotic neurons [19]. DNA demethylation in adult neurons, previously considered as rare if not impossible, has increasingly been found to impact on learned behaviour, neurotoxicity, cognition, CNS development and psychopathology [19,69,70,71,72]. Neurodegenerative diseases can cause both neurological and neuropsychological disabilities, including cognitive disorders in memory and learning, which may be related to the onset and progression of these devastating diseases. Based on the emerging role of active DNA demethylation and its possible association with mitochondrial oxidative metabolism in post-mitotic neurons, we here attempt to propose two potential therapeutic strategies for neurodegenerative diseases with respect to DNA demethylation both globally (reprogramming therapy) and in a gene-specific manner (mitohormesis therapy and gene-targeting approach using programmable Cas9- or transcription activator-like effector (TALE)-TET or DNMT fusion protein tools) (Figure 3).

The rationale of reprogramming therapy in adult neurons is to “reset” progressively degenerative neurons in the adult brain to a “stemness” status and expect that reactivation of a set of “stemness” genes would drive autonomous events to form fresh epigenetic marks and a functional neuronal gene network for recovering some critical brain functions. A similar idea has been proposed for drug discovery for new psychopharmacological agents [73,74]. Overexpressing “stemness” genes, such as developmental transcription factors *OCT4*, *SOX2* and *KLF4*, is widely adopted in reprogramming induced pluripotent stem (iPS) cells, although this is not explicitly practical in clinical therapy [73]. Instead, DNA methylation inhibitors (Table 2) have attracted significant attention because 5-azacytidine, a cytosine analogue to inhibit DNA methylation, was shown to potently reprogram differentiated cells [75]. Non-nucleoside inhibitors such as siRNA, antisense (MG98) and miRNA that directly down-regulate the DNNT expression can be also considered as “non-toxic” demethylating agents because they do not need to be incorporated into DNA [76]. In addition, HDAC inhibitors such as the aforementioned TSA or valproic acid may indirectly induce DNA demethylation by remodelling chromatin to an “opening” state; thus, HDAC inhibitors can be considered as an alternative [73]. The reprogramming therapy may have a number of inherent advantages over cell-replacement strategies such as stem cell therapy. For example, *in situ* resetting of neurons would not result in loss of the adult brain network; thus, in principle, the neuronal connectivity acquired through life-long experiences would be well preserved [74].

One of the major challenges in the use of inhibitors of DNA methyltransferases as therapeutic agents in post-mitotic neurons is the fact that most of the available DNA methylation inhibitors are ineffective at inducing demethylation in non-mitotic cells. This is largely due to their activity of suicide inhibition of DNMT as cytosine analogue; hence they require DNA synthesis to induce a reduction in DNA methylation. As mentioned above, post-mitotic neurons are non-dividing and thus lack DNA synthesis mechanisms, posing a severe barrier to such an approach. Nevertheless, several agents that directly reduce the affinity of DNMT in this category may have an encouraging future, in particular procainamide and RG108 (Table 2). Procainamide blocks the binding of DNMT to the substrate DNA, whereas RG108 interferes with DNMT by binding at its catalytic site [76]. Both possess potent DNA demethylation abilities but have less toxicity [77,78]. Procainamide has a long history of clinical use despite major concerns of cardiac arrhythmia and drug-induced lupus erythematosus due to its sodium channel-blocking properties [79]. RG108 has been shown to be efficacious in neuronal systems in preclinical cell and animal models for DNA demethylation and neuroprotective actions [80]. Non-nucleoside inhibitors such as siRNA, antisense (MG98) and miRNA that directly down-regulate DNNT expression can also be considered as “non-mitotic” and “non-toxic” demethylating agents because they do not need to be incorporated into DNA [76]. Among these, MG98 has been evaluated in phase I and II clinical trials for solid and hematopoietic malignancies; however, little clinical significance has been shown [81,82,83]. This perhaps attributes to the highly proliferative nature of these tumours, which may “dilute” its demethylating efficacy; in contrast, post-mitotic neurons are non-dividing and thus would be more suitable for this type of approach.

**Table 2:** DNA methylation inhibitors as possible therapies for neurodegenerative diseases.

Agent Name	Mechanism	Clinical Trials [73]	Concerns and Notes [79]
5-azacytidine	Cytosine analogue, suicide inhibitor of Dnmt	Clinically Tested: YES	Hematological malignancies, dose-limiting toxicity; Covalent DNA-protein trapping
		FDA-approved: YES	
		Crosses BBB: NO	
5-aza-2'-deoxycytidine (Decitabine, 5-azadC)	Cytosine analogue, suicide inhibitor of Dnmt	Clinically Tested: YES	Same as above
		FDA-approved: YES	
		Crosses BBB: NO	
Procainamide (Pronestyl)	Acts on Dnmt to reduce its affinity, non-nucleoside, blocks sodium channels and a specific inhibitor of Dnmt1 [77]	Clinically Tested: YES	Cardiac arrhythmia, sodium channel blocker; drug-induced lupus erythematosus
		FDA-approved: YES	
		Crosses BBB: YES	
(–)-epigallocatechin-3-*O*-gallate (EGCG)	Direct inhibition of Dnmt by reducing its affinity, non-nucleoside	Clinically Tested: YES	Strong topoisomerase inhibitor; should not be used by pregnant women because of increased risk of neonatal leukaemia and childhood malignant CNS tumours
		FDA-approved: NO	
		Crosses BBB: YES	
RG108	Direct inhibition of Dnmt by reducing its affinity, non-nucleoside and a specific inhibitor of Dnmt1 [78]	Clinically Tested: NO	Low concentration results in significant demethylation of genomic DNA without any detectable toxicity; preclinical for cancer chemotherapy and ALS therapy
		FDA-approved: NO	
		Crosses BBB: UNKNOWN	
Zebularine	Cytosine analogue	Clinically Tested: NO	Can be used orally; Lower toxicity than 5-azaC
		FDA-approved: NO	
		Crosses BBB: NO	
Hydralazine	Cytosine analogue	Clinically Tested: YES	Sympathetic stimulation of the heart; used successfully for myelodysplastic syndrome as a DNA methylation inhibitor
		FDA-approved: YES	
		Crosses BBB: NO	

BBB, blood-brain barrier.

Both cytosine methylation enzymes DNMT and TET methylcytosine dioxygenases work in multiprotein complexes and thus their global methylating and demethylating activities can be regulated by interaction partners in the DNA methylation and/or demethylation processes. For example, USP7 forms a trimeric complex with DNMT1 and UHRF1 and modulates the enzymatic activity of DNMT1 on the UHRF1 platform [84,85,86,87,88]. We and our colleagues have also shown that the methyl-CpG binding glycosylase MBD4 interacts with and recruits USP7 to heterochromatic foci, where it physically associates with UHRF1 and DNMT1, implicating it as an additional factor that can potentially regulate DNMT1 activity [89]. In terms of TET regulation, a CXXC-type zinc finger domain protein, IDAX, has been demonstrated to interact directly with the catalytic domain of TET2 to negatively regulate TET2 protein expression, which is sensitive to caspase activation and hence regulates TET enzymatic activity [90]. The *N*-termini of TET1 and TET3 contain the CXXC domain because their vertebral ancestors did not undergo chromosomal inversion like TET2 [91,92]. Notably, the CXXC domains of TET1 and TET3 can also regulate their expression and catalytic activity in a similar way and therefore control the levels of 5mC and 5hmC [90,93]. Consistently, in differentiated cells, global DNA demethylation was provoked by overexpression of the TET1 catalytic domain but not full-length TET1 that contains the CXXC domain [94], suggesting that the CXXC regulation may also work in non-dividing post-mitotic neurons. In addition, a number of other factors regulate DNMT and TET activity via chromatin recruitment or remodeling, such as HDACs and O-linked *N*-acetylglucosamine (O-GlcNAc) transferase (Ogt) [95,96,97]. These factors may have a crucial role in regulating DNMT and/or TET activity and thus modulate global DNA methylation levels in an oxidation-independent manner. Therefore, the therapeutic approaches targeting these factors may be useful to decrease DNMT enzyme activity or increase TET enzyme activity, stimulating the process of active demethylation.

Alternatively, metabolic reprogramming approaches may provide very useful therapeutic strategies for treating neurodegenerative diseases. In principle, such approaches may include cellular metabolic optimization by providing appropriate levels of mitochondrial biosynthetic capacity and energy production as well as balancing of the redox status [98]. In the context of the possible pathways of active DNA methylation, it would be rationale to consider stimulation of mitochondrial biogenesis and activity to increase the availability of metabolites involved in TET dioxygenase activity (αKG), decrease the availability of inhibitory metabolites (succinate, fumarate), limit the recycling of *S*-adenosyl homocysteine, or decrease the availability of SAM. One possible way would be to target the isocitrate dehydrogenases IDH1 and IDH2, which catalyse the conversion of isocitrate to αKG for an appropriate cellular redox balance [98]. In addition, the TET dioxygenases and HIF1α can be the subject of development of therapeutic strategies. However, one major concern of metabolic reprogramming is the potential for detrimental effects on normal metabolism that also depends on these pathways [99]. Perhaps more accurate measurement of specific metabolic dependencies of these potential gene targets is required for minimizing the oncogenic or other harmful effects on normal tissues. Several fluorescence-based sensors/reporters have been developed to measure metabolite concentrations and/or levels of ROS in the nanomolar range as well as in different organs and cellular compartments [36,100]. For example, HyPer, a chimeric peroxidase sensor, has the potential to indicate intracellular H_2_O_2_ levels in specific subcellular regions [36,100].

In contrast, mitohormesis therapy in post-mitotic neurons is based on a number of recent studies in which low levels of ROS have been shown to initiate protective molecular responses to prevent further cellular damage [37,101,102]. Mitochondrial ROS may act as signalling molecules to participate in insulin, growth factor, AP-1 and NF-κB cytosol pathways [36,101,102,103], which are believed to converge at the transcriptional level in the nucleus to result in protective stress defence [101]. We speculate that a number of neuroprotective and neuronal activity-responsive genes, which are inactivated during the onset and progression of neurodegeneration, would be gradually modified by repressive epigenetic marks, in particular DNA methylation; thereby perpetually inactivated during the onset and progression of neurodegeneration. The beneficial effects of ROS stress may specifically or non-specifically reactivate these protective genes by triggering yet unknown defence mechanisms in adult neurons. Active DNA demethylation may be one possible mechanism directly involved in, or at least that indirectly assists in, mitohormesis-based therapy; for example, the ROS-induced gene transcription itself may promote DNA demethylation at promoter regions, which allows the binding of transcriptional factors and primes the demethylated sites for subsequent inductions. However, this is still speculative as the nexus of active DNA demethylation and mitochondrial oxidative metabolism in post-mitotic neurons is just emerging and the majority of evidence relates to cytosol signalling. It remains to be established whether these cytosol signalling pathways can converge at the transcription of specific neuroprotective genes by triggering active DNA demethylation and what would be the optimal medical treatments and interventions, including dose of drug, stages of diseases and types of neurons, concerns of side effects or their possible combinations to maximise therapeutic effects as well as minimise toxicity. Recently, versatile genome editing technologies, such as fusion of the TET1 hydroxylase catalytic domain and engineered TALE or the RNA-guided endonuclease Cas9 from microbial type II clustered regularly interspaced short palindromic repeats (CRISPR), have enabled targeted DNA demethylation and activation of endogenous genes [104,105,106]. In principle, this should also work for specific activation of neuroprotective genes in post-mitotic neurons and/or for the selective repression of endogenous neurotoxic genes in an alternative combination with the DNMT catalytic domain (Figure 3). The study of crosstalk between active DNA demethylation and mitochondrial oxidative metabolism, its dynamic regulation, and pharmacological intervention in a tissue-selective manner, such as in post-mitotic neurons, would be very valuable for the development of therapeutic strategies in neurodegenerative diseases.
